# The complete mitochondrial genome of *Moricella rufonota* Rohwer, 1916 (Hymenoptera: Tenthredinidae) and phylogenetic analysis

**DOI:** 10.1080/23802359.2021.1920859

**Published:** 2021-07-12

**Authors:** Beibei Tan, Meicai Wei, Gengyun Niu

**Affiliations:** College of Life Sciences, Jiangxi Normal University, Nanchang, PR China

**Keywords:** Mitochondrial genome, phylogenetic analysis, Tenthredinidae

## Abstract

We sequenced the complete mitochondrial genome of *Moricella rufonota* Rohwer, 1916 (Tenthredinidae: Nematinae). The mitogenome is 15,731 bp in length with an A + T content of 81.9%, 37 typical animal mitochondrial genes, and a 386 bp control region. All the 13 protein-coding genes initiate with a typical ATN and end with TAA. The *trnI*(+)-*trnQ*(−)-*trnM*(+) cluster rearranged as *trnM*(+)-*trnQ*(−)-*trnI*(+) cluster, and the *trnW*(+)-*trnC*(−)-*trnY*(−) cluster rearranged as *trnC*(+)-*trnW*(+)-*trnY*(−) cluster. Phylogenetic analysis confirmed that the Nematinae is the basal lineage of Tenthredinidae, and *Moricella rufonota* is the basal lineage of Nematinae.

*Moricella rufonota* (Rohwer 1916) belongs to Dineurini, a tribe of Nematinae, Tenthredinidae (Wei and Nie 1998). It is an economically important leaf-chewing pest of Cinnamomum camphora (Linn.) Presl. Here, we reported the characterization of the complete mitogenome of *M. rufonota*. The complete mitogenome provides valuable information at the genomic level that can be utilized to sustain bioresources to deepen understanding of Nematinae.

Samples of *M. rufonota* (CSCS-Hym-MC0068) were collected from Xinting Village, Jiulong Wetland, Lishui, Zhejiang Province (28.402N, 119.828 E) in March 2018. The specimen was deposited at the Asia Sawfly Museum, Nanchang (ASMN) (Meicai Wei, weimc@126.com) under the voucher number CSCS-Hym-MC0068. Genomic DNA was prepared in 150 bp paired-end libraries, tagged, and analyzed with the high-throughput Illumina Hiseq 4000 platform. DNA sequences were assembled using MitoZ (Meng et al. 2019) and Geneious Prime version 2019.2.1 (Biomatters Ltd., Auckland, New Zealand). Sequences were annotated by the MITOS web server (Bernt et al. 2013). The sequences were multiply aligned using MAFFT method in the TranslatorX server (Abascal et al. 2010). The phylogenetic tree was reconstructed using maximum-likelihood (ML) estimate in the IQ-TREE webserver (Trifinopoulos et al. 2016).

A total of 93,836,266 raw reads were assembled by MitoZ. A sequence of 15,914 bp was yield, with *trnQ* absent. The *trnQ* was assembled by using *trnM* as a reference sequence (coverage was 22,028). The above-obtained sequence was thoroughly examined by reassembly using *Amauronematus saliciphagus* (unpublished) and *Hemichroa major* (unpublished) as reference sequences (coverage were 23,902 and 36,016, respectively). The control region was assembled by using *trnQ* and *trnI* as references. The length is 386 bp, contains two repeated regions of length 133 bp. The CG content was 22.8%, which is similar to *Hemichroa major* (unpublished). They are both the basal lineages of Nematinae (Prous 2014).

The complete mitochondrial genome of *M. rufonota* contains 37 genes and a 386 bp control region. Most of which are located in J-strand, except for the four protein-coding genes (PCGs) (*nad5*, *nad4*, *nad4L*, and *nad1*), two rRNA (*rrnL* and *rrnS*), and seven tRNA genes (*trnE*, *trnY*, *trnF*, *trnH*, *trnP*, *trnL2*, and *trnV*). Compared with the putative ancestral gene arrangement of insects (Boore 1999), the *trnI*(+)-*trnQ*(−)-*trnM*(+) cluster is rearranged as *trnM*(+)-*trnQ*(−)-*trnI*(+) cluster, in agreement with *Analcellicampa xanthosoma* (Niu et al. 2019). The *trnW*(+)-*trnC*(−)-*trnY*(−) cluster is rearranged as *trnC*(+)-*trnW*(+)-*trnY*(−) cluster, which is the first reported in Symphyta. The overall base composition is 43.2% A, 7.6% G, 10.5% C, and 38.7% T. All the 13 PCGs initiate with ATN, among which three genes (*atp8*, *nad5*, and *nad4l*) start with ATT; whereas five genes (*cox1*, *nad2*, *nad3*, *nad6*, and *nad5*) initiate with ATA; and five genes (*cox2*, *atp6*, *cox3*, *nad4*, and *cob*) start with ATG, and all 13 PCGs use TAA as the stop codon. The 22 tRNAs genes vary from 64 to 73 bp in length, and all the tRNAs form a classical clover-leaf secondary structure except for *trnS1*. The length of *rrnL* and *rrnS* are 1346 and 908 bp, respectively.

Phylogenetic analysis demonstrates that *M. rufonota* + (*A. xanthosoma* + *M. pruni*) form a monophyletic clade, representing Nematinae *s. lat.* This monophyly forms a sister group (Tenthredininae + Allantinae) + (*Sinopoppia nigroflagella* (Fenusinae + Blennocampinae)). The internal relationship among the Nematinae is still plagued by sparse sampling. All related files have been uploaded to Science Data Bank (http://www.scidb.cn/s/pfE773m) (Figure 1).

**Figure 1.**
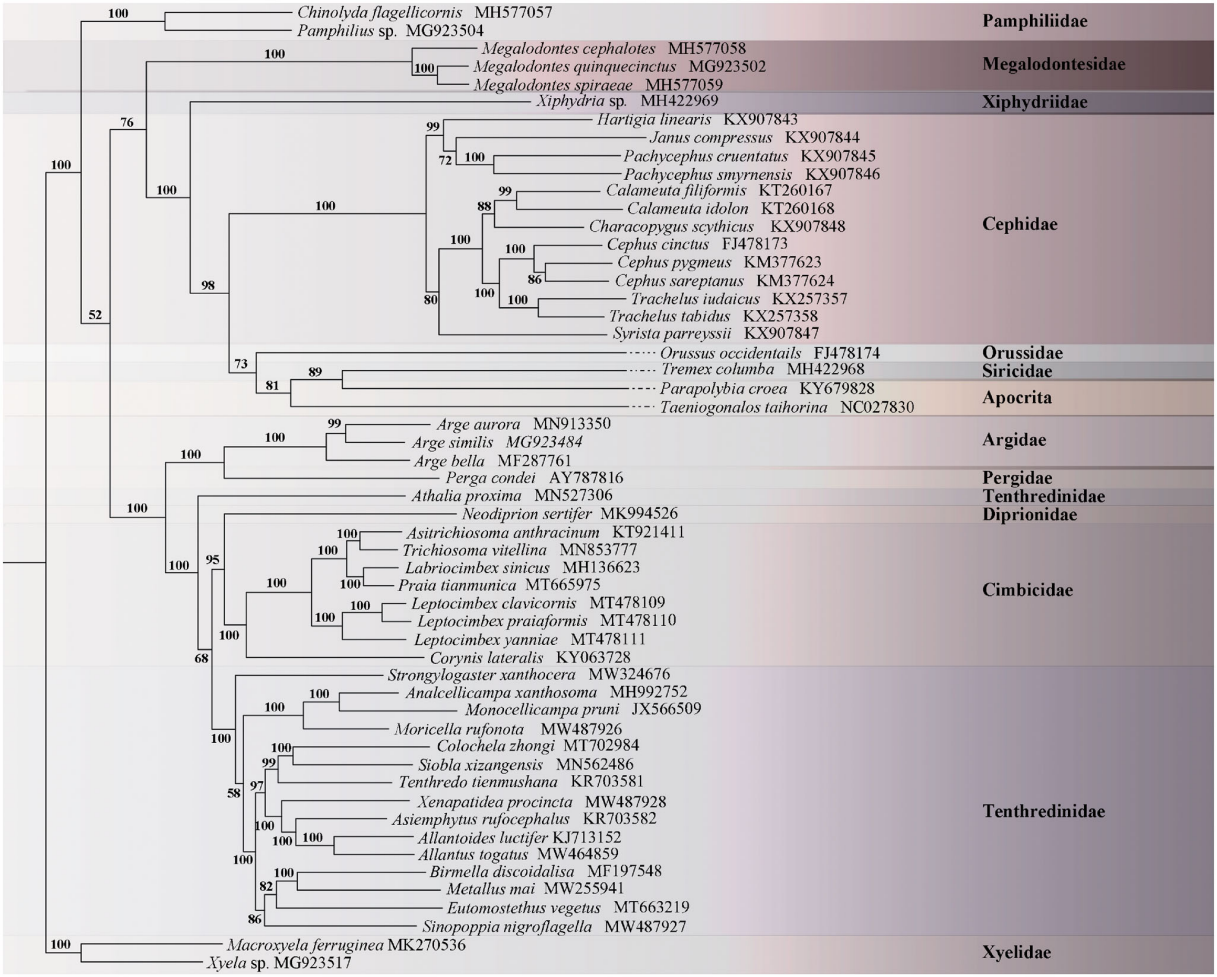
ML phylogeny base on protein-coding sequences of 54 species. Numbers at the left of nodes are bootstrap support value. The accession number of each species is indicated after the Latin name.

## Data Availability

The genome sequence data that support the findings of this study are openly available in GenBank of NCBI at [https://www. ncbi nlm nih gov] (https://www.ncbi. nlm nih gov/) under the accession number MW487926. The associated BioProject, SRA, and BioSample numbers are PRJNA692335, SRR13451118, and SAMN17320329.
